# Breakup Dynamics of Semi-dilute Polymer Solutions in a Microfluidic Flow-focusing Device

**DOI:** 10.3390/mi11040406

**Published:** 2020-04-14

**Authors:** Chun-Dong Xue, Xiao-Dong Chen, Yong-Jiang Li, Guo-Qing Hu, Tun Cao, Kai-Rong Qin

**Affiliations:** 1 School of Optoelectronic Engineering and Instrumentation Science, Dalian University of Technology, Dalian 116024, China; xuechundong@dlut.edu.cn (C.-D.X.); yongjiangli@dlut.edu.cn (Y.-J.L.); 2 School of Aerospace Engineering, Beijing Institute of Technology, Beijing 100081, China; xiaodong.chen@bit.edu.cn; 3 Department of Engineering Mechanics, Zhejiang University, Hangzhou 310027, China; ghu@zju.edu.cn

**Keywords:** droplet microfluidics, breakup dynamics, filament thinning, non-Newtonian fluids, flow-focusing device, extensional flow, semi-dilute polymer solutions

## Abstract

Droplet microfluidics involving non-Newtonian fluids is of great importance in both fundamental mechanisms and practical applications. In the present study, breakup dynamics in droplet generation of semi-dilute polymer solutions in a microfluidic flow-focusing device were experimentally investigated. We found that the filament thinning experiences a transition from a flow-driven to a capillary-driven regime, analogous to that of purely elastic fluids, while the highly elevated viscosity and complex network structures in the semi-dilute polymer solutions induce the breakup stages with a smaller power-law exponent and extensional relaxation time. It is elucidated that the elevated viscosity of the semi-dilute solution decelerates filament thinning in the flow-driven regime and the incomplete stretch of polymer molecules results in the smaller extensional relaxation time in the capillary-driven regime. These results extend the understanding of breakup dynamics in droplet generation of non-Newtonian fluids and provide guidance for microfluidic synthesis applications involving dense polymeric fluids.

## 1. Introduction

Droplet microfluidics has been one of the fastest growing areas of microfluidics in the past two decades, with far-reaching applications [1,2,3,4,5,6]. Microdroplets provide isolated micro-scale compartments for versatile functions in chemical, biological, medical, and material sciences [1,2,3,4,7,8,9,10,11,12]. Precise control of droplet size is a prerequisite to establish a uniform microenvironment, to ensure homogeneous composition, and to deliver accurate dosing of a drug or chemical reactants in these scenarios [9,10,13]. As non-Newtonian fluids are ubiquitous in nature and industry, non-Newtonian droplets are frequently encountered in practical processes including pharmaceutical synthesis, cell encapsulation, drug screening and delivery [14,15,16]. Understanding of non-Newtonian droplet generation is imperative in both fundamental mechanisms and practical applications.

Polymer solutions often exhibit non-Newtonian properties, i.e., elasticity, shear-thinning, or both. They have been frequently used as the model media in the laboratory due to their facile accessibility and easy-to-adjust rheology. Previous studies have demonstrated that a small amount of polymer added to a Newtonian fluid markedly affects the fluid rheology and, successively, the dynamics of breakup of the fluid threads in macroscopic experiments [17,18,19]. This kind of breakup can be observed in the experiments of capillary breakup extensional rheometry (CaBER), which has been developed as a novel method for rheology measurements of complex fluids [20,21,22]. Differing from the quiescent outer phase of free surface flows, the continuous phase fluid in droplet-based microfluidic devices deforms the interface of the two immiscible liquids and promotes interfacial instabilities [23,24,25]. Microfluidic devices with flow-focusing configuration are commonly used to generate microdroplets [23,26]. Typically, two immiscible liquids flow coaxially into separate channels with the continuous fluid flowing on both sides of the dispersed fluid, after which the resulting elongation-dominated flow field in the continuous liquid drives the dispersed liquid into a thin filament that breaks into separated droplets. The generation process becomes complicated when the dispersed phase is a non-Newtonian fluid, in which the delicate balance of capillary, inertial and viscous forces is influenced by extra factors such as the elastic stress [27,28,29].

The breakup dynamics of viscoelastic droplet generation in flow-focusing microchannels has been experimentally investigated by several groups. Blended with a small quantity of linear polymers, e.g., polyethylene oxide (PEO), pure water can become a viscoelastic fluid without shear-thinning. On the whole, the breakup dynamics of the elastic filament can be divided into a flow-driven regime and a capillary-driven regime based on transition time [30,31]. Initially, the thread head advances and restricts the flow of continuous fluid, resulting in an increase of the hydrostatic pressure upstream [32]. The increased pressure mainly triggers a fast necking of filament, forming the flow-driven regime in which the minimum thread width scales with the remaining time as a power-law relation [30,33,34]. The distribution of the accumulated pressure upstream then changes as the filament extends. The necking force decreases while the stretching force increases. Polymers in the dispersed thread are highly stretched while the elastic force starts to dominate and the necking enters into the later capillary-driven regime [31,35]. At the later stage, the thread thinning shows an exponential decay over time [30,31,33,34,35,36]. The exponential decay allows measurements of both the extensional relaxation time and the steady extensional viscosity [31,33,35]. In addition, the molecular weights of polymer and the dimensions of the microchannel have been reported to impose effects on the dynamics of filament breakup [31,32,33,35,37].

It is noted that most of the existing studies consider dilute polymer solutions, while more concentrated polymer solutions are frequently encounter in practical applications [5,14,15,16], which often exhibit non-Newtonian properties more than elasticity. Although several studies have also examined the effect of shear-thinning on filament breakup [38,39], the breakup behavior of dense polymer solutions exhibiting both elasticity and shear-thinning has rarely been addressed in microfluidic devices. In the present study, the breakup dynamics of semi-dilute polymer solutions in a flow-focusing microchannel are highlighted. The breakup morphology of semi-dilute PEO solutions is first characterized by comparing with those of the dilute and Newtonian counterparts. Filament thinning is then quantified by tracking the evolution of the minimum thread width, by which the flow-driven and capillary-driven regimes are identified. The characteristics in the breakup of the semi-dilute solutions are finally clarified by analyzing the two regimes in detail.

## 2. Materials and Methods

### 2.1. Fluids

The continuous and dispersed phase fluids were the olive oil and the aqueous solution of PEO (Sigma-Aldrich, Shanghai, China), respectively. The molecular weight of PEO was *M*_w_ = 2 M·Da. PEO solutions were prepared by adding PEO powders in deionized water (Milli-Q, 18 MΩ·cm) and blending it sufficiently using a shaker with a rate of 60/min for 1–2 days. Unlike previous studies focusing on dilute regimes, i.e., *c*/c^*^ < 1, semi-dilute solutions were focused on in this study, i.e., *c*/c^*^ = 2.5–10. The dilute cases, i.e., *c*/c^*^ = 0.1–1, were also considered for comparison. Here, c^*^ is the overlap concentration calculated as c^*^ = 3*M*_w_/4π*R*_g_^3^*N*_A_ = 1000 ppm (parts per million), where *N_A_* is the Avogadro constant and *R_g_* = 0.02*M*_w_^0.58^ = 90 nm is the gyration radius of a single polymer molecule [40]. The density (*ρ*_c_) and viscosity (*η*_c_) of olive oil were 0.92 g/cm^3^ and 78 mPa·s, respectively. The interfacial tension (σ) of the continuous and droplet phase was measured as 20.3 ± 0.08 mN/m using the pendant droplet method (Theta, Attension Inc., Goteborg, Switzerland).

### 2.2. Rheological Characterization

**Figure 1:**
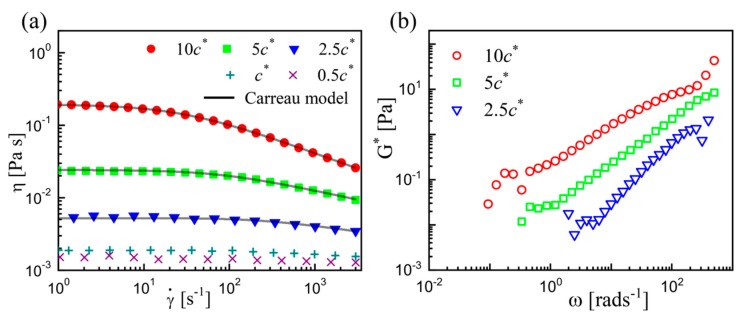
Rheological characterization of PEO solutions. (**a**) The measured viscosities *η* as a function of shear rate $\dot{\gamma }$ at different concentrations. The solid curves at *c* > c^*^ are fitted by the modified Carreau model. The overlap concentration is c^*^ = 1000 ppm. (**b**) The measured complex moduli at different concentrations.

The rheological properties of PEO solutions were measured using a rotational rheometer with cone-plate (50 mm, 0.3 rad) (Physica MCR302, Anton Paar GmbH, Glaz, Austria). The viscosities *η* were measured at shear rates of 0.1–3000 s^−1^ (Figure 1a), and the complex moduli (G^*^) as a function of angular frequency (*ω*) was measured in dynamic oscillatory experiments with a constant strain of 4% (Figure 1b). All the measurements were conducted at 25 °C maintained by a Peltier stage. The shear-rate-dependent viscosities and the complex moduli indicate the coexistence of shear-thinning and elasticity. Meanwhile, both the shear-thinning and the elasticity enhance with the PEO concentration *c*.

To quantify the rheological properties of the semi-dilute solutions, a modified Carreau model [41,42] was used to fit the measured data:

(1) $$\eta (\dot{\gamma })=\eta_{0}{\left[1+{\left(\lambda_{C}\cdot \dot{\gamma }\right)}^{a}\right]}^{(-d)/a}$$

where *η_0_* is the zero shear viscosity, *λ*_C_ is the characteristic time for the fluid, *d* is the negative of the power-law slope, and *a* is the fitting parameter introduced by Yasuda et al. [41] Note that the *η_0_* is viscosity at $\dot{\gamma }$ ~ *O*(10^−1^) in the actual measurement. Here, *d* = 0 signifies no shear-thinning. The *d*-value increases as PEO concentration increases, indicating the increased shear-thinning effect. The characteristic time *λ*_C_ corresponds to the critical shear rate ($\dot{\gamma }$) at which the *η* begins to decrease [41], which can be deemed as the relaxation time of the polymeric fluid in our measurements. All the fitting parameters are listed in Table 1.

For comparison, values for Zimm relaxation time (*λ*_Z_) and reptation time (*λ*_R_) are provided. The Zimm relaxation time is calculated based on the Zimm model [33,43,44], which can describe the relaxation of dilute solutions well. The *λ*_Z_ values were estimated by

(2) $$\;\lambda_{Z}=0.463\frac{[\eta ]M_{w}\eta_{s}}{N_{A}k_{B}T}$$

where [*η*] = 0.072*M*_w_^0.65^ is the intrinsic viscosity of PEO solution, *η*_s_ is the solvent viscosity, *N*_A_ is the Avogadro’s number, *k*_B_ is the Boltzmann’s constant, and *T* is the absolute temperature. The prefactor 0.463 accounts for the solvent quality and the excluded volume parameter. Accordingly, *λ*_Z_ = 0.35 ms is obtained, independent of the polymer concentrations. In contrast, the reptation model takes the hydrodynamic interactions into account and can better depict the polymer dynamics in semi-dilute solutions [45,46,47]. The *λ*_R_ value shows a concentration dependence, i.e.,

(3) $$\lambda_{R}=\tau_{0}N^{3}/N_{e}\left(1\right)c^{3\left(1-v\right)/(3v-1)}$$

where *τ_0_* ≈ 0.2 ns is the monomer relaxation time at room temperature, *N* = *M*_w_/*M*_0_ is the number of Kuhn monomers per chain (*M*_0_ = 140 for PEO), *N*_e_(1) ≈ 14 is the number of Kuhn monomers in an entanglement strand in melts, and *v* = 0.588 is the solvent quality exponent. The estimated λ_R_ values agree well with the values of *λ*_C_ for the semi-dilute solutions, as shown in Table 1. The estimated values of *λ*_C_ were also compared with those measured by other approaches, i.e., CaBER (*λ*_1_) [21] and Dripping-onto-Substrate rheometry (DoSR) (*λ*_2_) [44]. The comparison suggests that our estimated relaxation time values accord with those reported in these works (Table 1).

### 2.3. Experimental Setup

**Figure 2:**
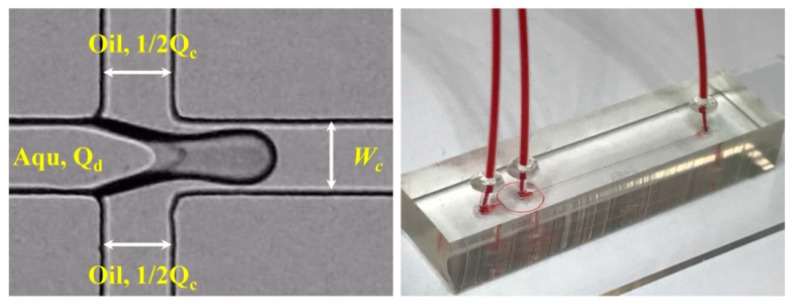
Channel configuration and assembled microchip. All the openings of the cross-slot channel are 50 μm. *Q*_c_ and *Q*_d_ represent the flow rates of the continuous phase and dispersed phase, respectively. Red ink was infused to visualize the channel structure in the assembled chip.

The flow-focusing configuration was employed in the present study, as shown in Figure 2. The main channel was 50 μm in both width and height (*W*_c_). All the openings of the cross-slot structure were 50 μm. The intermediate inlet was set to introduce dispersed phase fluid. The two identical side openings are for continuous phases, which are then merged into one main inlet for easy control in actual operation. The chip was fabricated in polydimethylsiloxane (PDMS), based on the standard soft-lithography technique as described previously [48,49]. Before the experiments, the chips were kept in an oven at 80 °C overnight to restore the material to its native hydrophobic condition. The square geometry and the hydrophobic properties were designed to minimize the three-dimensional effect as far as possible, allowing us to rationally analyze the droplet generation process just from the in-plane images [35].

The experiments were performed under an inverted microscope (Nikon Eclipse Ti, Nikon Instruments Inc., Tokyo, Japan). The fluids were introduced into the assembled chip by two syringe pumps (Pump 11 Elite, Harvard apparatus Inc., Holliston, Massachusetts, United States) with two 1 mL plastic syringes (BD, Becton Dickinson Inc., Franklin lakes, New Jersey, United States). The flow rates of continuous and dispersed fluid were *Q*_c_ = 20–160 μL/h and *Q*_d_ = 1.25–160 μL/h, respectively, and the flow rate ratios were *q* = *Q*_c_/*Q*_d_ = 1–16. For this range of control parameters, the Reynolds number of continuous phase *Re*_c_ = *ρ*_c_*W*_c_*U*_m_/*η*_c_ is 0.001–0.01 and the capillary number *Ca*_c_ = *η*_c_*U*_m_/σ is 0.009–0.07, where *U*_m_ = *Q*_c_/*W*_c_^2^ is the average velocity of continuous phase fluid. The low *Re*_c_ and *Ca*_c_ indicate that in the quasi-static state the inertial forces are much smaller than the viscous forces and the viscous forces are much smaller than the surface forces. Moreover, to evaluate the importance of elasticity in the dispersed phase, the Weissenberg number, defined as the ratio between the fluid relaxation time and the flow characteristic time, was also considered. In the focused semi-dilute cases, *Wi = 2λ_Z_Q_d_/W_c_^3^* = 0.02–24.9, suggesting that the elastic effect can dominate under most conditions.

The images of the droplet breakup process were recorded using a high-speed CCD camera (Phantom v7.3, Vision Research Inc., Birmingham, Alabama, United States) and the Phantom Camera Control software (PCC 2.14, Vision Research Inc., Birmingham, Alabama,, United States). Experimental videos were taken at 10^3^–10^4^ fps (frames per second). All of the experiments were conducted at the room temperature. The captured images were then processed and analyzed using the ImageJ software package (National Institute of Health, Bethesda, Maryland, USA). The maximum length error of image processing was estimated to be 4 μm, and the time error was less than the interval between two successive images, i.e., 100 μs.

## 3. Results and Discussion

### 3.1. Qualitative Observations

**Figure 3:**
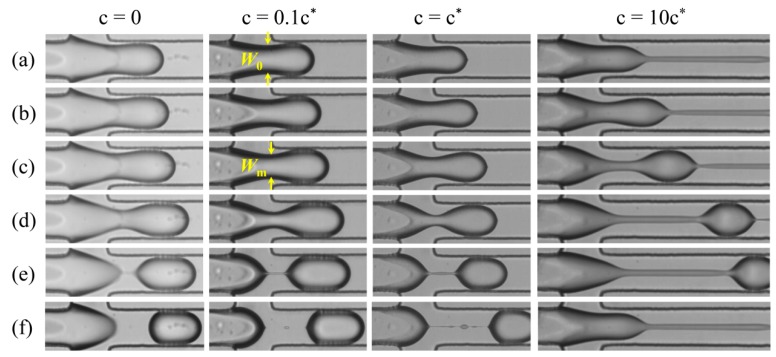
Sequences of images showing the thinning process of droplet generation for different solutions: (**a**) Initial regime; (**b**) *t*/*t*_b_ = 0.2; (**c**) *t*/*t*_b_ = 0.4; (**d**) *t*/*t*_b_ = 0.8; (**e**) *t*/*t*_b_ = 1; and (**f**) after breakup. Values of *t*_b_ for the four cases are 14.8, 16.8, 18.4 and 60 ms, respectively. *W*_0_ represents the initial thread width and *W*_m_ represents the minimum thread width at a certain instant. For all cases, *q* = 4 and *Q*_c_ = 40 μL/h.

The image sequences listed in Figure 3 demonstrate the thinning processes of the dispersed thread for different polymer solutions under the identical flow condition, i.e., *q* = 4 and *Q*_c_ = 40 μL/h. The case of pure water, i.e., *c* = 0, exhibits typical filament thinning and droplet generation of Newtonian fluid. Initially, the dispersed thread starts to elongate and neck after it is driven into the junction. The thread then thins quickly and forms a primary droplet connected to a thin filament. The thin filament finally breaks into a satellite droplet. To simplify the analysis, the breakup time *t*_b_ is defined as the instant when the filament is uniform and the satellite droplets have not yet formed, as shown in Figure 3e. These observations are consistent with previous experiments [31,37,50].

The polymeric cases exhibit different behavior at different polymer concentrations. The dilute solutions, i.e., *c* = 0.1c^*^ and c^*^, display behavior similar to the Newtonian counterpart. The dispersed fluid also develops into a thread that necks first and then breaks into a primary droplet and some tiny satellite droplets. The filament length and the breakup time in these polymeric cases are longer than those of the Newtonian counterpart, as previously reported [31,33]. Due to the low flow rate ratio *q*, the discrepancies here are not so large. Meanwhile, for the semi-dilute solution, i.e., *c* = 10c^*^, the thread thinning exhibits a very different behavior. During the early phase, the morphology is similar to that of the Newtonian counterpart. As time elapses, the filament further elongates but thins at a much lower rate. The filament elongation and breakup time *t*_b_ are much longer. After breakup, the filament destabilizes and its width becomes non-uniform, leading to the well-known bead-on-a-string that forms multiple satellite droplets successively [31,51]. This process occurs between two primary droplets downstream and is not shown in the image. Although the satellite droplets formed during filament breakup, the primary droplets on which we focused in the current study are highly uniform for all cases. Notably, the satellite droplets become larger as the PEO concentration increases or the flow rate of continuous phase increases, inevitably reducing the uniformity of the generated droplets. How to improve the uniformity of polymeric droplets is another issue that deserves to be addressed in the future.

### 3.2. Quantitative Characterization

The observations above give us an intuitive impression. To capture more details, the process of filament thinning was quantitatively characterized by tracking the evolution of minimum width of filament *W*_m_. The distinct interface of dispersed thread offers the chance to track the evolution of filament width. As denoted in Figure 3, the width at the instant when the thread starts to form is defined as the initial width *W*_0_, and this instant is defined as the initial time *t* = 0. As time elapses, the thread deforms to form a necked filament. The distance between the two points with zero curvature in the neck is defined as the minimum width of filament *W*_m_. As the thinning progresses, the measurement location of *W*_m_ shifts slightly but always maintains a location near the cross-slot. The time evolution of *W*_m_ is then extracted from the sequential images of individual thinning period. To ensure reliability, more than three measurements were carried out for each case.

**Figure 4:**
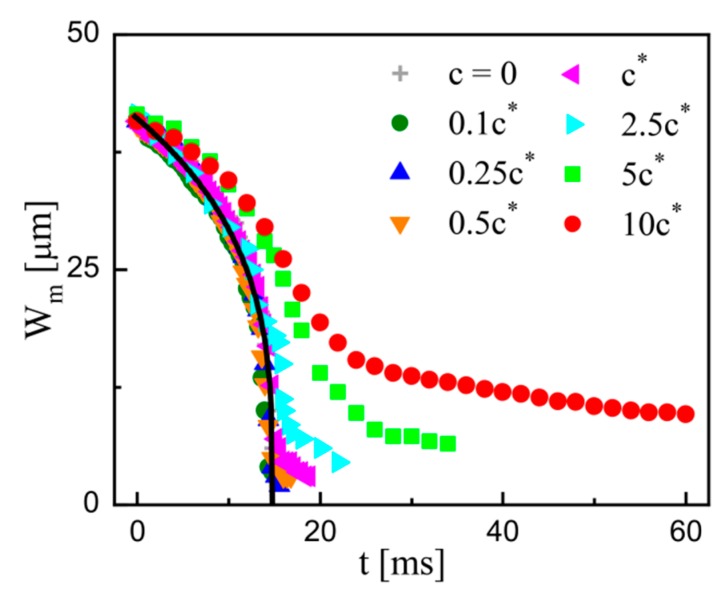
Time evolution of the minimum filament width *W*_m_ at different PEO concentrations. The black solid curve *W*_m_ ~ (14.8 − *t*)^0.32^ is the fit for the Newtonian case, i.e., *c* = 0. The flow conditions are identical.

Figure 4 shows the typical results of time-varied *W*_m_ under different conditions. The measured data are truncated at the breakup time *t_b_* when the width thread is uniform, and the satellite droplets have not formed yet. The result of pure water (*c* = 0) is provided as the Newtonian counterpart. As shown by the black solid curve in Figure 4, the power-law fit *W*_m_ ~ (14.8 − *t*)^0.32^ is consistent with previous microscopic experiments with Newtonian fluids [37,50]. In contrast, the polymeric cases display a different behavior. The decay is similar to that of the Newtonian counterpart in the early stages but deviates upwards later. The deviation enhances with the increasing concentration *c*, and the breakup time *t*_b_ increases. Moreover, the retardation of decay in the early stage can be noticed when the polymer concentration *c* increases to the semi-dilute regime (*c* > c^*^). This retardation also shows a positive dependence on the polymer concentration *c*. This will be further explained in combination with the fitting law in the next section.

The instant when the deviation starts can be defined as the transition time *t*_p_. For the viscoelastic filament, the thinning process is divided into a flow-driven stage and an elasto-capillary stage based on the transition time *t*_p_ [30,31], which can be captured by comparing the time-varied profiles of *W*_m_ between the polymeric fluid and the Newtonian counterpart [30,43]. As shown in Figure 4, it is valid for dilute solutions (*c* ≤ c^*^) but cannot be directly applied for semi-dilute solutions (*c* > c^*^) in the current study. Here, we propose a method to extract the values of *t*_p_ based on the temporal variation of the extensional strain rates in the filament thinning. The extensional strain rates can be calculated by [30,31,43,52]

(4) $$\dot{\varepsilon }=\frac{-2}{W_{m}}\frac{dW_{m}}{dt}$$

**Figure 5:**
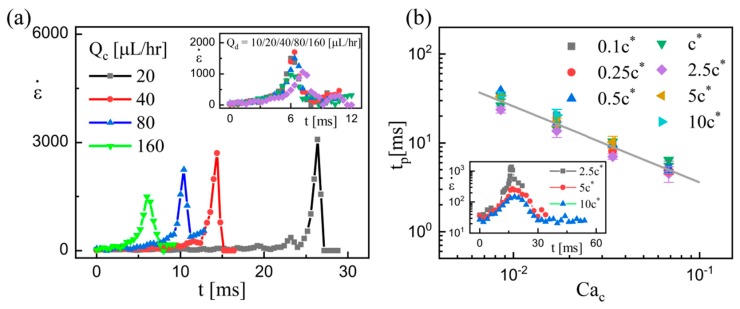
(**a**) Extensional strain rate $\dot{\varepsilon }$ as functions of the elapsed time at different *Q*_c_. The moment when the maximum $\dot{\varepsilon }$ occurs is regarded as the transition time *t*_p_. The PEO concentration *c* = c*. Inset: The time-varied $\dot{\varepsilon }$ at different *Q*_d_. (**b**) Values of transition time *t*_p_ as functions of capillary number *Ca*_c_. The straight line *t*_p_ ~ *Ca*_c_^−0.86^ is the best fit, and the error bars represent standard deviations. Inset: The time-varied $\dot{\varepsilon }$ at different *c*.

It is found that the values of $\dot{\varepsilon }$ increase initially and decrease afterwards as time elapses, and the two-staged characteristics are generic for all the examined solutions (Figure 5a). The moment when the maximum $\dot{\varepsilon }$ occurs can be reasonably regarded as the transition time *t*_p_, before which the elastic stress is negligible. The elastic stress becomes dominant only when the polymer molecules are stretched to a certain extent at the maximum $\dot{\varepsilon }$ [31,43].

Figure 5a shows the extensional strain rate $\dot{\varepsilon }$ as a function of the elapsed time at different flow rates of continuous phase *Q*_c_, and the variation of $\dot{\varepsilon }$ at various flow rates of dispersed phase *Q*_d_ (inset). It is clear that the transition time *t_p_* inversely depends on *Q*_c_ but is almost independent of *Q*_d_. Conceivably, the increasing *Q*_c_ can accelerate the rate of droplet generation, in which the required time for individual filament thinning deceases. The transition time *t*_p_ decreases as a consequence. In contrast, the increasing *Q*_d_ always enhances the droplet size but hardly affects the generation rate, thus imposing a negligible effect on *t*_p_. In addition, although the time evolution curves of *W*_m_ at different PEO concentrations are different (Figure 4), the transition time *t*_p_ varies slightly as the PEO concentration increases (inset in Figure 5b). By analyzing all the experimentally captured values of *t*_p_, the relation of *Q*_c_ and *t*_p_ can be established, as shown in Figure 5b. The dimensionless parameter *Ca*_c_ (proportional to *Q*_c_) is introduced to fit the relation *t*_p_ ~ *Ca*_c_^−0.86^.

**Figure 6:**
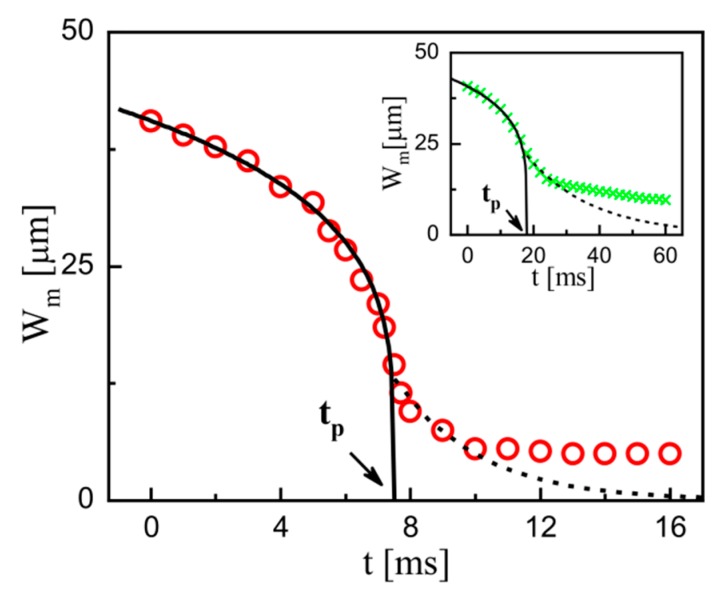
Typical fitting of the time-varied *W*_m_ at *c =* 2.5c^*^. The flow conditions are *q* = 8 and *Q_c_* = 80 μL/h. The fitting is a combination of *W*_m_ ~ (*t*_p_ − *t*)*^α^* (*α* = 0.24, solid curve) and *W*_m_ ~ exp[−*κ*(*t* − *t*_p_)] (*κ* = 0.39, dotted curve). The transition time *t*_p_ and breakup time *t*_b_ are 7.5 ms and 16 ms, respectively. Inset: The experimental data at *c =* 10c^*^and the corresponding fit. The flow conditions are *q* = 4 and *Q*_c_ = 40 μL/h. *t*_p_ and *t*_b_ are 18 ms and 60 ms, respectively. The fitting parameters are *α* = 0.21 and *β* = 0.048.

It is obvious that the filament thinning can be divided into two regimes based on the transition time *t*_p_ for all the examined PEO solutions (Figure 4). This two-stage feature is the same to the previous experiments for purely elastic fluids, in which a power-law function along with an exponential function are often used to fit the thinning process [30,33,43,44,53]. It is widely acknowledged that the power-law function characterizes the flow-driven regime, while the exponential function characterizes the capillary-driven regime in the viscoelastic cases. Here, a combination of the two functions is also tried to fit our measured data for the semi-dilute solutions. Figure 6 shows the typical fitting of the time-varied profile of *W*_m_ at *c* = 2.5c^*^, in which *W*_m_ ~ (*t*_p_ − *t*)^0.24^ and *W*_m_ ~ exp[−0.39(*t* − *t*_p_)] are separately denoted by the solid and dotted curves. For a denser solution (*c* = 10c^*^), the similar fit can be obtained (see inset in Figure 6). These results demonstrate that the combination of flow-driven regime and capillary-driven regime is applicable for all the examined PEO solutions. To unveil the potential effects of the other properties in semi-dilute polymer solutions, the two regimes are separately analyzed in detail.

### 3.3. Flow-Driven Regime

**Figure 7:**
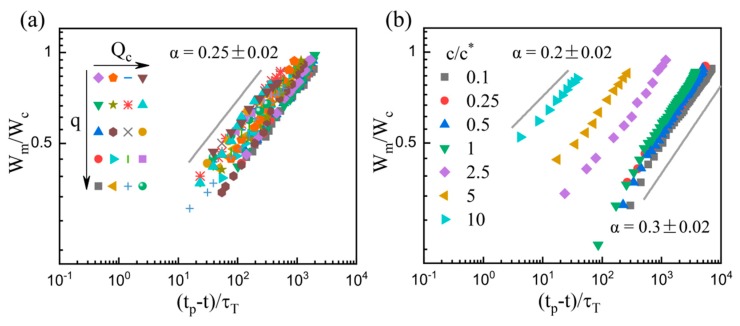
Plots of the dimensionless minimum width *W*_m_/*W*_c_ of the dispersed filament to the dimensionless remaining time (*t*_p_ − *t*)/*τ*_T_ for *c* = 2.5c* under different flow conditions (**a**) and for different concentrations (**b**). In (**a**), *q* = 1–16 and *Q*_c_ = 20–160 μL/h. In (**b**), *q* = 2 and *Q*_c_ = 40 μL/h. All grey lines are provided to guide the eye.

To describe the relation of the minimum filament width *W*_m_ to the remaining time (*t*_p_ − *t*) in the flow-driven regime, a power-law function was applied as follows:

(5) $$W_{m}/W_{c}=A{[(t_{p}-t)/\tau_{T}]}^{\alpha }$$

where *W*_c_ is the channel width and *τ*_T_ = *η*_d_*W*_c_/*σ is* the characteristic viscous time, and they are used to build the dimensionless quantity. Figure 7 shows the experimental data and the corresponding fits. Parameter *A* also shows a positive dependence on the flow rate of continuous phase and the viscosity of dispersed phase at *c* ≥ c^*^, i.e., *A* = 0.36*Ca*_c_^0.13^ and *A* = 1.36*Ca*_d_^0.33^, where *Ca*_d_ = *Ca*_c_(*η*_c_/*η*_d_), consistent with those in the Newtonian and dilute cases [30,50]. At a fixed concentration of *c* = 2.5c^*^, a power-law exponent *α =* 0.25 ± 0.02 is obtained, independent of the flow rate ratio *q* and the flow rate of continuous phase *Q*_c_ (Figure 7a). The constant exponent indicates a self-similar breakup process in the flow-driven regime in current configuration. The exponent*α* is approximately 1/4 and is smaller to the 2/3 or 1/2 for free liquid threads [43,44,54], reflecting the effects of the channel confinement and external flow. Moreover, the exponent *α* shows a dependence on the PEO concentration *c*, as illustrated in Figure 7b. For the dilute solutions with *c* ≤ c^*^, the constant exponent *α =* 0.30 ± 0.02, which is in consistent with *α =* 0.36, was obtained for the purely elastic fluids in the similar flow-focusing microchannel [30]. At the highest concentration of *c* = 10c^*^, the exponent *α =* 0.20 ± 0.02. Meanwhile, the exponent *α* decreases with the increasing *c* in the semi-dilute cases (*c* > c^*^).

The decreased exponent *α* indicates the deceleration of filament thinning in the flow-driven regime, which is caused by the elevated viscosity of the dispersed thread. The rheological measurement shows that the viscosity of polymer solution increases with increasing *c* (Figure 1a). In dilute cases, the increment is very limited, i.e., the viscosity at *c* = c^*^ is only two times that of pure water. As *c* further increases, a dramatic increase occurs, e.g., the viscosity at *c* = 10c^*^ is greater than that of pure water by two orders of magnitude (Table 1). The highly elevated viscosity of the dispersed thread in semi-dilute cases consequentially inhibits filament thinning. Besides, the bulk rheology also shows that the shear-thinning effect (reflected by *d*-value) enhances with increasing *c* (Table 1). In contrast to the effect of elevated viscosity, shear-thinning should exert an effect to accelerate the breakup (by improving the exponent *α*). However, our results report the decreased exponent *α*, suggesting the screened effect of shear-thinning in the current experiments, which agrees with the observations of previous experiments for purely shear-thinning fluids [38,39].

### 3.4. Capillary-Driven Regime

In the capillary-driven regime with *t* > *t*_p_, the relationship between dimensionless minimum filament width *W*_m_/*W*_c_ to dimensionless remaining time (*t* − *t*_p_)/*τ*_T_ can be depicted by an exponential function:

(6) $$W_{m}/W_{c}\propto \exp[\kappa (t-t_{p})/\tau_{T}]$$

**Figure 8:**
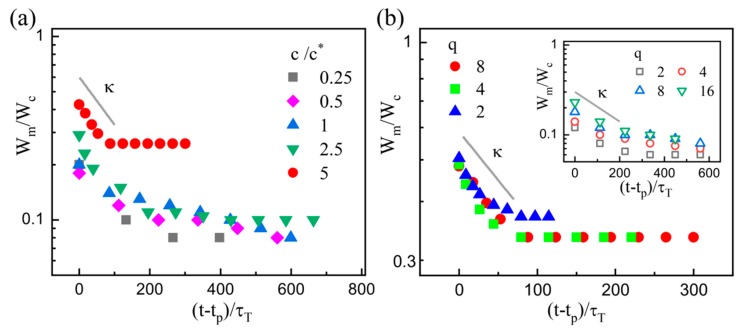
Plot of the dimensionless minimum width *W*_m_/*W*_c_ of the dispersed filament to the dimensionless shifted time (*t* − *t*_p_)/*τ*_T_ at (**a**) various *c*; and (**b**) *c* = 5c^*^. In (**a**), *q* = 8 and *Q*_c_ = 80 μL/h. In (**b**), *Q*_c_ = 80 μL/h. Inset: *c* = 0.5c^*^ and *Q*_d_ = 10 μL/h.

Figure 8a shows the experimental data at various PEO concentrations, which suggests that the exponential decay is genetic in both dilute and semi-dilute cases. The results are also in line with those for purely elastic fluids in previous experiments [30,33,35,43]. Moreover, the values of parameter *κ* barely depend on the flow conditions, as demonstrated in Figure 8b. Recalling the previous theory, the exponential regime for high viscosity elastic fluids based on the balance between elastic and capillary forces follows the function [19,43,54]

(7) $$W_{m}/W_{c}\propto \exp[-(t-t_{p})/3\lambda_{E}]$$

where *λ*_E_ is the extensional relaxation time. The relation *λ*_E_ = −*τ*_T_/(3*κ*) can be derived and the values of *λ*_E_ for all the examined solutions can be calculated. The latent distinction between dilute and semi-dilute cases could be acquired by the analysis of *λ*_E_.

**Figure 9:**
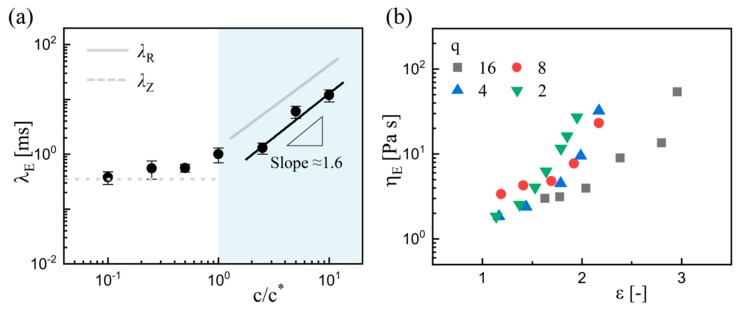
Results of extensional relaxation time *λ*_E_ and extensional viscosity *η*_E_. (**a**) Variation of extensional relaxation time values *λ*_E_ with PEO concentrations. The grey line represents the predicted values for reptation time λ_R_, and the grey dashed line represents the predicted values for the Zimm relaxation time λ_Z_. (**b**) Evolution of extensional viscosity *η*_E_ with Hencky strain ε at *c* = 5c^*^.

Figure 9a shows the estimated values of extensional relaxation time *λ_E_* for all the examined solutions. For the dilute solutions, i.e., *c* ≤ c^*^, the *λ_E_* values are several times greater than the Zimm relaxation time *λ*_Z_ (dotted line in Figure 9a), which agrees with the findings of previous similar experiments [33]. Due to the microchannel confinements and the continuous phase flow, these estimated *λ*_E_ values are generally smaller than those captured in the pinch-off experiments of free surface fluid [33,43,44]. For the semi-dilute solutions, i.e., *c* > c^*^, the estimated *λ*_E_ shows similar *c*-dependence (*λ*_E_ ~ *c*^1.6^) with the reptation time *λ*_R_ (*λ*_R_ ~ *c*^1.5^), while the *λ*_E_ values are obviously smaller than *λ*_R_. These smaller *λ*_E_ values indicate the incomplete stretch of polymer molecules in the capillary-driven regime. This coincides with the result that the coil-stretch transition becomes milder for more concentrated polymer solutions in both macroscopic and microscopic extensional flows [44,55]. Moreover, the accumulated strain or Hencky strain ε can be calculated as ε = 2ln(*W*_0_/*W*_t_), [35,52] where *W*_t_ denotes the time-varied *W*_m_. It is thus found that the maximum strain ε_max_ = [4.5–6] at *c* = 0.1c^*^, while ε_max_ = [1–2] at *c* = 10c^*^. This contrast supports the claim of incomplete stretch in the semi-dilute solutions.

It is speculated that the incomplete stretch is due to the dense network structures in the semi-dilute solutions. According to the blob theory [45,47], each PEO molecule forms a blob when *c* is below c^*^ and all the blobs do not contact with each other. As *c* increases above c^*^, blobs overlap with each other to form the networks and the network structures become denser with increasing polymer concentrations. For dilute polymer solutions, individual polymer blobs can be highly stretched in the filament. For semi-dilute polymer solutions, the networks are stretched as a whole and the individual polymer chains are just partially stretched, resulting in the lower strain ε, as stated above.

Furthermore, the extensional viscosity is introduced due to the polymer stretch. The extensional viscosity *η*_E_ is finally examined, which is calculated using the formula [30,44]

(8) $$\;\eta_{E}=\frac{\sigma }{\dot{\varepsilon }W_{t}}=\frac{\sigma }{-2dW_{m}/dt}$$

Figure 9b illustrates the variation of extensional viscosity *η*_E_ with an increasing Hencky strain ε at *c* = 5c^*^. The extensional viscosity *η*_E_ increases with the strain ε, indicating the obvious strain hardening that has been reported in both macroscopic [53,54] and other microscopic experiments [30,31,33]. The absolute values of *η*_E_ are two to three orders of magnitude greater than their zero-shear viscosity *η*_0_, and this enhancement contributes to the delayed breakup. In the final stage, the filament further elongates and thins nearly linearly with time (Figure 4 and Figure 6). The last regime allows the measurement of terminal steady extensional viscosity, which would be reached before all the chains are completely stretched out [44,56]. It is predicted that the final regime is maintained by the competitive effect between the continued stretch of the polymer chains and the drainage of the dispersed filament. This mechanism needs to be further validated by studying the polymer dynamics in the filament in the future work.

## 4. Conclusions

In summary, we experimentally investigate the filament breakup in droplet generation of semi-dilute PEO solutions in a flow-focusing microchannel. The breakup morphology of semi-dilute PEO solutions is analogous to that in dilute cases, which falls into a universal temporal profile in combination with a flow-driven regime and a capillary-driven regime. The transition time *t*_p_ dividing the two regimes is extracted based on the temporal variation of the extensional strain rates. The power-law exponent *α* characterizing the flow-driven regime in semi-dilute cases is smaller than that in dilute cases and decreases with increasing PEO concentration. It is ascertained that the highly elevated viscosity of the semi-dilute solution decelerates the filament thinning in the flow-driven regime while the accelerating effect of shear-thinning is screened. The capillary-driven regime is analyzed based on the extensional relaxation time *λ*_E_ and the extensional viscosity *η*_E_. The extensional relaxation time *λ*_E_ being less than the reptation time *λ*_R_ indicates the incomplete stretch of polymer molecules in the capillary-driven regime. The extensional viscosity *η*_E_ shows obvious strain hardening, and the significant enhancement of *η*_E_ contributes to the delayed breakup. These results extend the understanding of breakup dynamics in droplet generation of non-Newtonian fluids and could provide guidance for microfluidic synthesis applications involving semi-dilute polymer solutions.

## Figures and Tables

**Table 1:** The fitting parameters of the Carreau model and three kinds of relaxation time for comparison.

*c*/c^*^	*η*_0_ (mPa s)	*d*	*a*	*λ*_C_ (ms)	*λ*_R_ (ms)	*λ*_1_ (ms)	*λ*_2_ (ms)
2.5	5.2	0.16	1.3	4.2	5.2	-	9
5	24	0.25	1.1	12	15	19.4	12
10	193	0.43	0.96	35	42	86	26
